# Generation of New Neurons in Dorsal Root Ganglia in Adult Rats after Peripheral Nerve Crush Injury

**DOI:** 10.1155/2015/860546

**Published:** 2015-02-03

**Authors:** Luisa Muratori, Giulia Ronchi, Stefania Raimondo, Stefano Geuna, Maria Giuseppina Giacobini-Robecchi, Michele Fornaro

**Affiliations:** ^1^Department of Clinical and Biological Sciences, University of Turin, 10043 Orbassano, Italy; ^2^Neuroscience Institute “Cavalieri Ottolenghi” (NICO), 10043 Orbassano, Italy; ^3^Department of Anatomy, Chicago College of Osteopathic Medicine, Midwestern University, 555 31st Street, Downers Grove, IL 60515, USA

## Abstract

The evidence of neurons generated *ex novo* in sensory ganglia of adult animals is still debated. In the present study, we investigated, using high resolution light microscopy and stereological analysis, the changes in the number of neurons in dorsal root ganglia after 30 days from a crush lesion of the rat brachial plexus terminal branches. Results showed, as expected, a relevant hypertrophy of dorsal root ganglion neurons. In addition, we reported, for the first time in the literature, that neuronal hypertrophy was accompanied by massive neuronal hyperplasia leading to a 42% increase of the number of primary sensory neurons. Moreover, ultrastructural analyses on sensory neurons showed that there was not a relevant neuronal loss as a consequence of the nerve injury. The evidence of BrdU-immunopositive neurons and neural progenitors labeled with Ki67, nanog, nestin, and sox-2 confirmed the stereological evidence of posttraumatic neurogenesis in dorsal root ganglia. Analysis of morphological changes following axonal damage in addition to immunofluorescence characterization of cell phenotype suggested that the neuronal precursors which give rise to the newly generated neurons could be represented by satellite glial cells that actively proliferate after the lesion and are able to differentiate toward the neuronal lineage.

## 1. Introduction

The dorsal root ganglia (DRG) are located along the dorsal spinal roots and are surrounded by a connective capsule that isolates this cluster of neurons. The location and the connective capsule define the DRG as an isolated peripheral pool of neuronal bodies that, for this reason, are easily identifiable and represent a valid model for the study of permanent neurons. Therefore, the absence of postnatal cell migration from or to the DRG makes it a particularly suitable model for the study of adult neurogenesis due to the presence of a stem cell niche within the ganglia [1–9].

The generation of new neurons in both central and peripheral adult nervous systems is well acknowledged today [6, 10–15]. Although it is still a controversial matter, for decades several groups have collected data suggesting that, in different animal species, DRG may undergo a progressive age-dependent increase in neuron number [2, 8, 16–19].

Recently, it has been demonstrated* in vitro* that adult rat DRG and trigeminal ganglia explants are able to give rise to neurospheres that can differentiate into neurons and glia [6, 13, 20]. Moreover, an* in vivo* study demonstrated that, as a consequence of peripheral nerve injury (crush lesion or axonotmesis), DRG neurons undergo adaptive changes [21, 22] enabling them to respond and recover from injury [23–26]. Finally, evidence of satellite glial cells proliferation was demonstrated in adult rats DRG after capsaicin injection [27].

In the present study, we investigated whether the sequence of events that follow peripheral axon damage also included a change in the number of DRG neurons assessed by a means of accurate and unbiased stereological counts. We considered that the exceptional stimulus represented by massive nerve regeneration, which is characterized by the presence of supernumerary axons distal to the lesion site [28], may retrogradely stimulate plasticity in the corresponding neurons of DRG. For our experiments, we adopted the nerve crush lesion paradigm using a nonserrated clamp [29], which causes axonotmesis without interrupting epineurial continuity and thus posttraumatic axonal regeneration occurs without requiring surgical repair of the nerve.

Our stereological results indicated that a relevant increase in neurons number occurred in the DRG belonging to the brachial plexus during the first month after crush injury of the four main terminal plexus branches. The presence of BrdU-immunopositive neurons expressing the neural progenitor markers supported the stereological evidence of posttraumatic neurogenesis and suggested that the precursor cell population which gives rise to the new-generated neurons may be represented by satellite glial cells.

To test our hypothesis on the role of satellite glial cells (SGCs), we characterized the cellular morphological changes that follow the crush injury using immunofluorescence and transmission electron microscopy (TEM) analyses. Our results supported the view that the neuronal precursors are represented by SGCs that actively proliferate after the lesion and are able to differentiate toward the neuronal lineage.

## 2. Materials and Methods

### 2.1. Animals and Surgical Procedure

Adult female Wistar rats (Charles River Laboratories, Milan, Italy) weighing approximately 190–220 g were used for this study (*N* = 25). All procedures were performed in accordance with the Ethics Committee and the European Communities Council Directive of November 24, 1986 (86/609/EEC). Adequate measures were taken to minimize pain and discomfort taking into account human endpoints for animal suffering and distress.

All surgical procedures were carried out under deep anesthesia obtained with tiletamine + zolazepam (Zoletil) i.m. (3 mg/kg). The median, ulnar, radial, and musculocutaneous nerves of the left forelimb were approached from the axillary region to the elbow with a longitudinal skin access. Under operative microscope, nerves were carefully exposed from their origin at the brachial plexus until the elbow. The crush lesion was applied to each nerve using a nonserrated clamp [29]. Animal well-being assessment was carried out using careful animal surveillance to check for passive and active movements, automutilation, skin ulcers, and joint contracture, especially during early postoperative times.

### 2.2. Sample Collection

Rats were sacrificed by a lethal i.m. injection of tiletamine + zolazepam. The vertebral column was surgically dissected and the vertebral bodies were cut off and removed in order to reach the spinal cord. Briefly, using fine scissors, we accessed the vertebral canal performing a double cut on both sides of the vertebral bodies. Using the dorsal spinal roots as guides, the ganglia that participate in the formation of the brachial plexus (the last 4 cervical, C5–C8, and the first thoracic, T1) were identified and removed.

For the stereological analysis, T1 DRG from both sides were removed after 30 days from the crush injury and processed for the resin embedding procedure. DRG were removed also from 5 healthy rats from both sides, as control.

For the immunohistochemical analysis, animals underwent BrdU injections (see Section 2.6) and DRG were harvested at different time points: 1, 3, 5, 7, 10, 15, and 30 days from the crush injury and processed for the paraffin embedding procedure. DRG removed from healthy rats were used as control.

### 2.3. Resin Embedding

DRG were fixed by immediate immersion in 2.5% glutaraldehyde for 2 h, washed in Sorensen phosphate buffer 0.1 M (pH 7.4) with 1.5% sucrose, and postfixed in 2% osmium tetroxide for 2 h. After dehydration in ethanol, samples were cleared in propylene oxide and embedded in Glauerts' embedding mixture of resins consisting in equal parts of Araldite M and Araldite Harter, HY 964 (Merck, Darmstadt, Germany), containing 0.5% of the plasticizer dibutyl phthalate and 1-2% of the accelerator 964, DY 064 (Merck, Darmstadt, Germany).

### 2.4. Stereology Analysis for Sensory Neurons Number

For stereological analysis, only T1 ganglion from both sides was analyzed since it predominantly contributes, in terms of number of sensory fibers, to the crush injured peripheral nerves. All of DRG, randomly oriented, were cut into serial semithin sections (2.5 *µ*m) using an Ultracut UCT ultramicrotome (Leica, Wetzlar, Germany) and stained with 1% toluidine blue.

The physical disector stereological analysis [30] was performed on T1 DRG divided into 4 experimental groups (*n* = 5/group): (1) left T1s harvested 30 days after the nerve crush lesion (CRUSH); (2) right T1s taken from the same animal of group 1 used as internal control (INTERNAL CTRL); and (3) and (4) left and right T1-DRG taken from healthy animals that did not undergo the crush lesion (NORMAL LEFT and NORMAL RIGHT).

**Figure 1:**
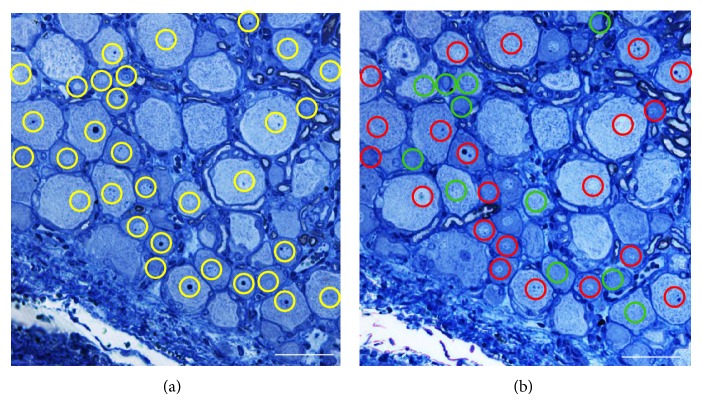
Example of the toluidine blue stained micrographs of reference (a) and counting (b) sections from which neurons numbers are estimated using the physical disector. All nuclear profiles are recognized in the reference section (yellow circles); nuclear profiles that do not appear in the counting section are counted (green circles). The nuclei of cells that appear in both the counting and reference sections are not counted (red circles). Bars = 50 *µ*m.

Four/six disector pairs (depending on the size and the orientation of the DRG) were selected by systematic random sampling [30] from each DRG, setting the distance between consecutive disector pairs at 100 *µ*m. The reference section was taken at 5 *µ*m from the counting section. The determination of neuron number was based on the identification of the top of the nucleus; each nucleus in the reference section was identified, marked, and carefully recognized in the counting section under high-resolution light microscope observation by blinded observers (Figures 1(a) and 1(b)). Only nuclear profiles that were observable in the reference section but not in the counting section (thus suggesting the nucleus ended in the thickness between the pair of sections analyzed) were counted (Figure 1(b)) and the average density (*N*_*v*_) was calculated. Then, the reference volume (*V*_ref_) of the entire ganglion was estimated using the Cavalieri principle; the fibrous portion of the hilum was not included in the reference volume. Finally, the total number (*N*) of the DRG neurons was calculated as *N* = *V*_ref_ × *N*_*v*_ [31]. The precision of the estimates was evaluated by calculating the coefficient of error (CE) as described by Schmitz [32] and the sampling scheme was designed in order to keep the CE below 0.10, which assures enough accuracy for neuromorphological studies [33].

### 2.5. Morphometric Analysis for Sensory Neurons Size

Left T1-CRUSH DRG and left T1-CTRL DRG were processed for morphometric analysis in order to evaluate the size of the sensory neurons. Morphometric analysis was performed on the same cells counted for the stereological analysis. For this purpose, serial semithin section previously used for the stereology was considered and the neurons area was measured using the unbiased point counting method with a DM4000B microscope equipped with a DFC320 digital camera and an IM50 image manager system (Leica Microsystems, Germany). Areas were converted into diameters and cells size distribution was obtained.

### 2.6. BrdU-Treatment

**Figure 2:**
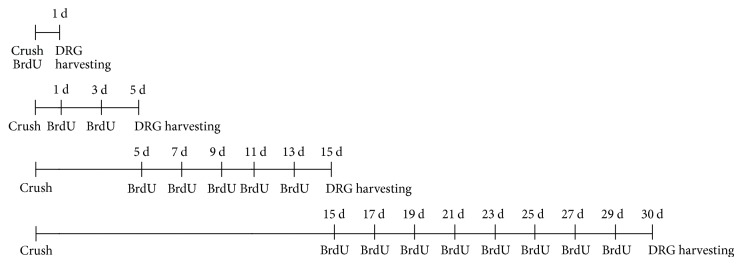
Experimental design of BrdU administration.

For the qualitative and quantitative evaluation of BrdU incorporation, animals were injected intraperitoneally with 5-bromo-2-deoxyuridine (BrdU, Sigma, St. Louis, MO, 50 mg/kg, made from 10 mM BrdU dissolved in 7 mM NaOH) diluted in PBS. The experimental design of BrdU administration is illustrated in Figure 2. Animals scarified 1 day after crush lesion were subjected to one single BrdU injection on the same day of the surgery. Animals scarified 5 days after the crush lesion were subjected to BrdU injection at days 1 and 3, while rats scarified after 15 days were subjected to BrdU injection every other day starting from the 5th day after the injury. Finally, rats scarified 30 days after crush lesion were subjected to BrdU injection every other day starting from the 15th day after the injury. This protocol was used in order to prevent an overlapping between the different time points, thus allowing us to predict the BrdU incorporation rate in a determined time window. DRG were harvested 1, 5, 15, and 30 days after the crush injury. T1 DRG were used for BrdU quantification, whereas C5–C8 DRG were used for qualitative evaluation.

### 2.7. Stereology for BrdU Quantification

Stereological analysis was performed on T1 DRG embedded in paraffin and cut into serial sections (10 *µ*m) to assess the number of BrdU-positive cells, which was counted by blinded observers with a variation of the physical disector method adapted to confocal laser microscopy [34].

BrdU-positive cells were counted [30] as neuronal and nonneuronal cells based on their morphological features. As an additional criterion, nestin immunopositivity (see Section 2.10) was used to distinguish small neurons from satellite cells in case of doubts. Briefly, confocal *z*-stacks of five random samples for each DRG were taken and considered for count. For each sample, the counting/reference pair of sections was randomly selected at 3 *µ*m distance from each other. The determination of BrdU-positive cells was based on the identification of the top of the nucleus; each nucleus in the reference level was identified and only nuclear profiles that were observable in the counting level but not in the reference section were counted. Finally, to predict the daily rate of cells which incorporate BrdU in the different time windows, the number of BrdU-positive cells was divided by the days of BrdU treatment.

### 2.8. Statistics

Statistical analysis was performed using both one-way analysis of variance (ANOVA) test and *t*-test for morphometric data. Parametric tests were adopted assuming that sample mean values for all estimated parameters present a normal distribution. Values are expressed as mean ± standard deviation (SD). The level of significance was set at *P* ≤ 0.05 (^*^), *P* ≤ 0.01 (^**^), and *P* ≤ 0.001 (^***^). All statistical tests were performed using SPSS software.

### 2.9. High-Resolution Light Microscopy and Electron Microscopy Analysis

For light and electron microscopy analysis, DRG samples of control rats (CTRL) and 1 day and 5 days after crush injury (CRUSH) were embedded in resin (see Section 2.3). Transversal cross sections of 2.5 *µ*m (light microscopy) and 70 nm (electron microscopy) were obtained from the DRG using an Ultracut UCT ultramicrotome (Leica, Wetzlar, Germany). Sections for light microscopy were then stained with toluidine blue and images were taken with a DM4000B microscope equipped with a DFC320 digital camera and an IM50 image manager system (Leica Microsystems, Germany). Sections for electron microscopy were stained with uranyl acetate and lead citrate and examined by a JEM-1010 transmission electron microscope (JEOL, Tokyo, Japan) equipped with a Mega-View-III digital camera and a Soft-Imaging-System (SIS, Münster, Germany) for the computerized acquisition of the images.

### 2.10. Immunofluorescence

Samples were fixed in 4% paraformaldehyde for 2 h, washed in a solution of 0.2% glycine in 0.1 M phosphate buffer (pH 7.2), dehydrated, and embedded in paraffin. Sections were cut with thicknesses of 10 *μ*m by a RM2135 microtome (Leica Microsystems, Wetzlar, Germany).

For BrdU staining, rehydrated sections were incubated with 2 N HCl (in PBS, 0.1% triton X100 solution, 15 min at room temperature), rinsed in PBS, and neutralized with 0.1 M sodium tetraborate. Sections were then incubated with monoclonal antibody anti-BrdU.

For all the other staining techniques, sections were permeabilized, blocked (0.1% triton X-100, 10% normal goat serum (NGS)/0.1% NaN_3_, 1 h), and processed for an immunohistochemical protocol. See Table 1 for the list of primary antibodies used.

Sections were incubated overnight in primary antibody or, in case of double immunofluorescence experiments, in a mixture of primary antibodies and visualized using a solution containing the appropriate secondary antibody/ies: goat anti-mouse IgG Alexa-Fluor-488-conjugated (1 : 200, Molecular Probes, Eugene, Oregon), rabbit anti-goat IgG Alexa-Fluor-488-conjugated (1 : 200, Molecular Probes, Eugene, Oregon), and CY3-conjugated anti-rabbit IgG (dilution 1 : 400, Dako, Milan, Italy). The samples were finally mounted with a Dako fluorescent mounting medium and analyzed by a LSM 510 confocal laser microscopy system (Zeiss, Jena, Germany).

## 3. Results

### 3.1. Stereological Evaluation of DRG Sensory Neuronal Number and Size

The effect of the nerve crush lesion applied to the radial, ulnar, median, and musculocutaneous nerves on the thoracic T1 DRG sensory neurons was investigated 30 days after nerve damage by quantitative evaluation of DRG neurons total number. For this purpose, the physical disector stereological method, which deals better with the difficulty of counting the number of neurons (considering their different cell sizes) within the ganglia, was applied.

In order to avoid artifacts, as, for instance, tissue shrinkage, thus guaranteeing a more precise stereological analysis, the physical disector method was performed on semithin sections that underwent the embedding procedure utilized for electron microscopy (see Section 2). In addition, for a more complete evaluation of differences within the cell population of the two sets of ganglia (CRUSH and NORMAL LEFT), in consideration of a physiological variability between the pair (left and right) of the same ganglia, the quantification of the neuronal population of the two groups was compared to the contralateral ganglia, which means the right T1s taken from the same animal that underwent crush lesion (INTERNAL CONTROL) and the right T1s from control (NORMAL RIGHT), respectively.

**Figure 3:**
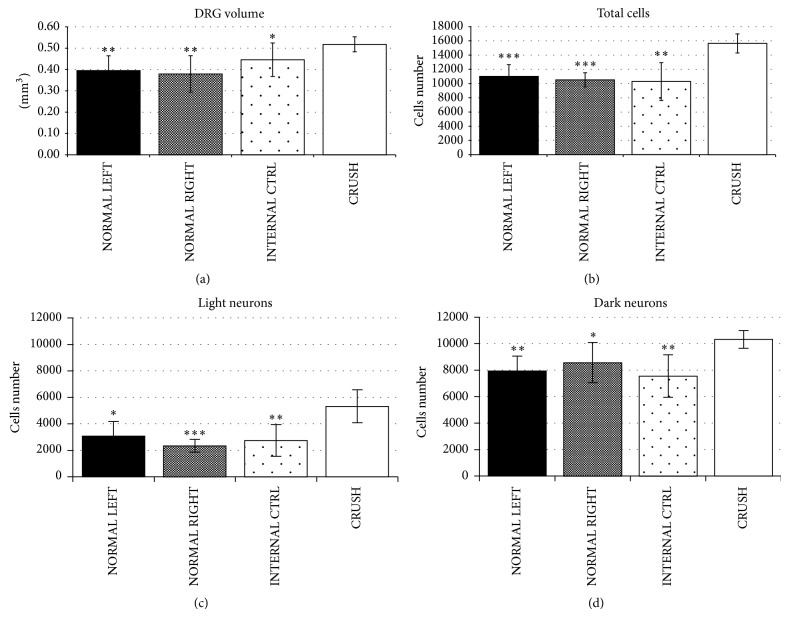
(a) T1s affected by crush lesion showed an evident hypertrophy compared to controls. (b) Physical disector stereological analysis was applied to T1s ganglia. The overpopulation of neurons was distributed among the light and large neurons (c-d). The data were analyzed using both ANOVA and *t*-test analyses. ^*^ *P* ≤ 0.05; ^**^ *P* ≤ 0.01; ^***^ *P* ≤ 0.001 versus CRUSH group.

The analysis of the entire DRG volume showed an evident hypertrophy after 30 days from the crush injury compared to all the three control groups (Figure 3(a)). For the total cell number count, in order to discriminate neurons that needed to be included in the count, the quantitative analysis was carried out by blinded observers. The physical disector stereological method demonstrated a significant increase of neurons within hypertrophic crushed T1s (CRUSH: 15642 ± 1347) compared to controls (NORMAL LEFT: 11006 ± 1649; NORMAL RIGHT: 10512 ± 995; INTERNAL CTRL: 10285 ± 2650) (Figure 3(b)). A neuronal overexpansion of 42% was estimated for the DRG neuronal population in crushed animals compared to nonoperated animals. Moreover, as shown in Figures 3(c) and 3(d), the overpopulation of neurons was distributed among the two neuronal subpopulations: the light and the dark neurons.

**Figure 4:**
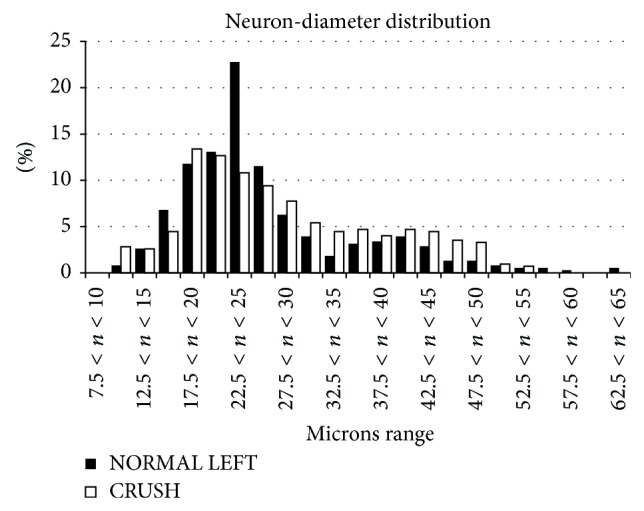
Diameter-frequency distribution of pooled T1 DRG neurons belonging to CRUSH and NORMAL LEFT groups. There is a small rightward shift in diameter-frequency distribution in animals that underwent crush injury.

Finally, we examined the changes in the size distribution of all sensory neurons in T1s after 30 days from the crush injury (Figure 4) observing a right shift (i.e., hypertrophy) in the neuron-diameter distribution of animals that underwent crush injury compared to controls.

### 3.2. Morphological Changes Occurring within DRG after Nerve Crush Lesion

**Figure 5:**
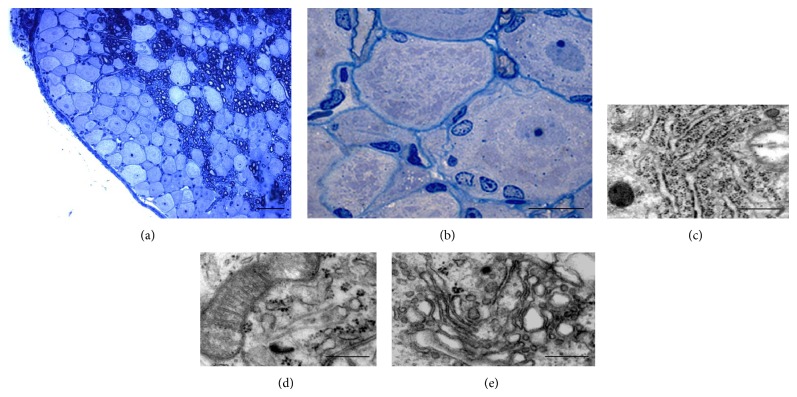
Resin-embedded 2.5 *µ*m semithin sections of crushed DRG at low (a) and high (b) magnification stained with toluidine blue. The ultrastructural analysis in electron microscopy shows that sensory neurons are particularly rich in organelles as RER (c), mitochondria (d), and Golgi apparatus (e). Scale bars: a = 50 *µ*m; b = 20 *µ*m; c–e = 0.5 *µ*m.

Morphological analysis showed that most of the neurons of the crush group appeared different compared to controls. The DRG neurons in crushed animals (Figures 5(a) and 5(b)) showed a nonhomogeneous cytoplasm aspect due to an organization of subcellular organelles and neurofilaments, typically seen associated with an increase in metabolic cellular activity. The ultrastructural analysis in electron microscopy showed neurons particularly rich in subcellular organelles such as rough endoplasmic reticulum (RER), mitochondria, Golgi apparatus, and many free ribosomes, with no signs of cell suffering as a consequence of the peripheral nerve lesion (Figures 5(c)–5(e)).

**Figure 6:**
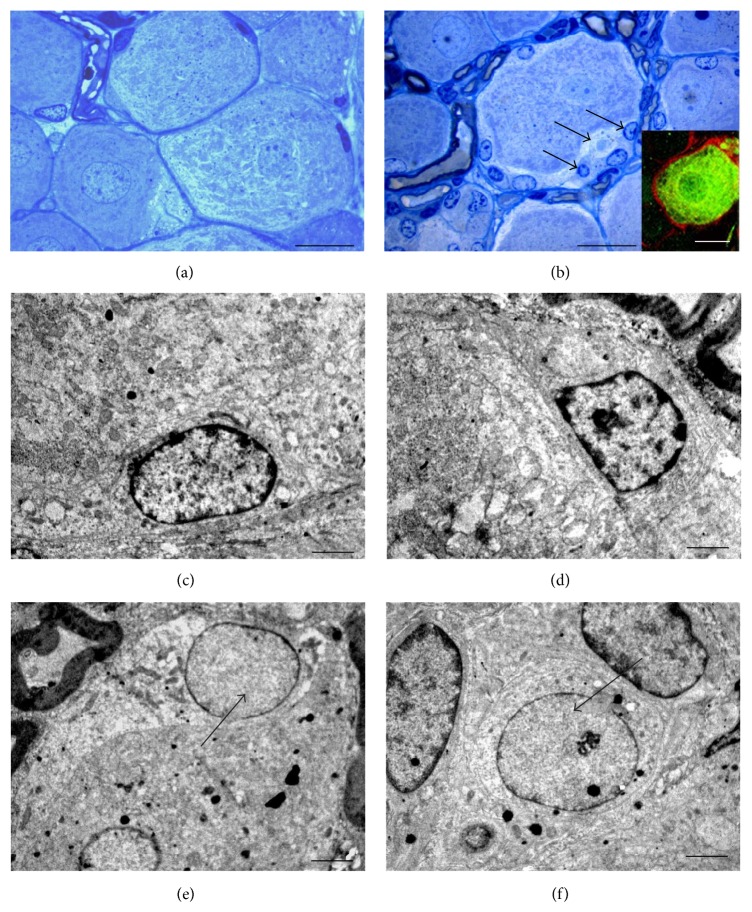
High magnification light microscopy pictures of toluidine blue stained semithin sections of controls (a) and DRG harvested 5 days after nerve crush lesion (b) in which many cells are seen surrounding neurons (b, arrows). A double fluorescence staining shows S100-positive cells (red) surrounding a neurofilament 200 KDa-positive (green) neuron (box in b). The ultrastructural analysis in electron microscopy shows at low magnification morphological features of glial cells (c, d) and neuronal-like cells (e, f, arrows). Scale bars: a, b = 20 *µ*m; c–f = 2 *µ*m.

Noteworthy, as a consequence of the crush lesion applied to the peripheral nerves, an increased population of cells with different structural and ultrastructural features appeared within DRG (Figure 6). The shape of neurons changed and many cells, not seen in controls (Figure 6(a)), were seen surrounding, as a clear crown, the neuronal profiles starting from day 1 after lesion (Figure 6(b)). Among the new population of cells, many different morphological features allowed the discrimination of different cell types. Some of these cells were immunopositive for the glial marker anti-S100 (box in Figure 6(b)). The morphological differences were investigated also in electron microscopy. Electron microscopy analysis showed a diffuse electron dense chromatin in the nucleus of some of these cells particularly evident close to the nuclear membrane and a dark cytoplasm rich in endoplasmic reticulum (Figures 6(c) and 6(d)). The same morphological characteristics were also seen in the Schwann cells surrounding fibers within the DRG, thus suggesting a glial phenotype.

The other cell profile showed a clear round nucleus with a barely observable chromatin and a nucleolus that was often detectable. The cytoplasm was usually clearer than the other cell type and poorer in cytoplasmic organelles (Figures 6(e) and 6(f)). These clear areas of cytoplasm showed the presence of neurofilaments. These morphological features suggested a less differentiated cell population compared to the one described above.

### 3.3. *In Vivo* New Neurons Identification

**Figure 7:**
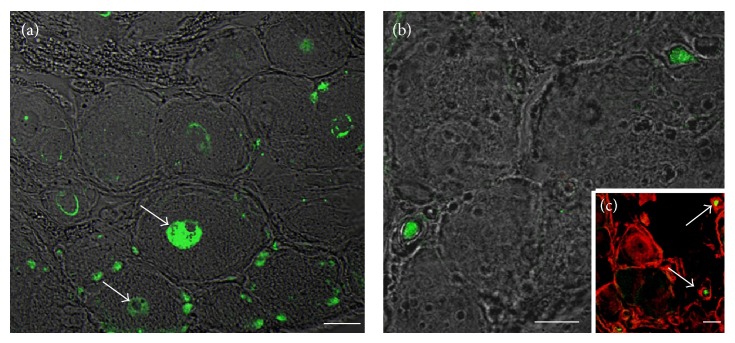
Immunofluorescence images of DRG after crush lesion and BrdU treatment for the qualitative evaluation. 15 days after crush lesion, many small cell nuclei surrounding ganglionic neurons as well as some nuclei belonging to sensory neurons are found to be BrdU-positive (a, arrows). Cells localized surrounding the ganglionic neurons are immunolabeled with Ki67, an endogenous marker for proliferation 3 days after crush lesion (b). A double labeling using anti-Ki67 (green) associated with anti-peripherin (red), 7 days after injury, shows a coexpression of the two markers in ganglionic neurons (c). Scale bars: a–c = 20 *µ*m.

**Figure 8:**
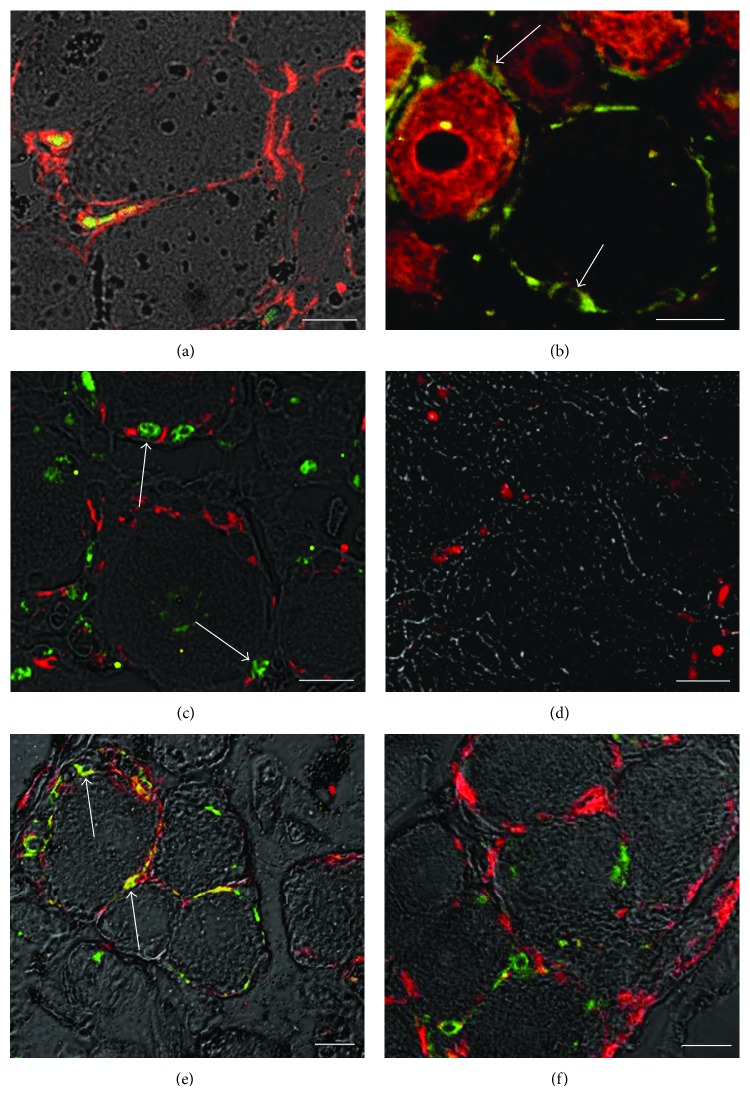
After 3 days from crush lesion, cells coexpressing nanog (red) and Ki67 (green) were found (a). A double immunofluorescence performed at day 5 after injury detects sox-2 positive cells (green) surrounding neurons, some of which are peripherin-positive (red) (b, arrows). At the same time point, the same cells are found coexpressing BrdU (green) and nestin (red) (c, arrows). After 7 days from crush lesion, anti-NeuroD is seen to be expressed in many nuclei surrounding the DRG neurons (d). Further characterization of this cell population was done using markers for both immature and mature glial cells. A double labeling using nestin (green) and the immature glial marker GFAP (red) shows that, at day 5 after injury, the two markers colocalize in some cells (e, arrows). However, no colocalization is observed in double labeling of nestin with S-100 (mature glia, in red) (f). Scale bars: a–f = 20 *µ*m.

The appearance of this new population of cells within DRG after crush lesion led to further investigations in which crush injured animals and control were inoculated* in vivo* with BrdU. For the qualitative evaluation, DRG C5-T1 were harvested and analyzed in immunohistochemistry at different time points after crush lesion (Figures 7 and 8).

After 15 days, many small BrdU-positive cells surrounding neurons were found; a clear BrdU labeling was indeed detected in nuclei belonging to ganglionic neurons (Figure 7(a)). None of the neurons in the control ganglia were found to express BrdU (data not shown). BrdU analysis was correlated to other markers endogenously expressed in proliferating cells, as, for instance, Ki67, which was found expressed in some cells that would seem to be surrounding large neurons 3 days after crush lesion (Figure 7(b)). As shown in Figure 7(c), after 7 days, we detected a coexpression of Ki67 and peripherin, a neuronal marker specifically expressed by the “small and light” neuronal subpopulation and ubiquitously expressed in young DRG neurons [35]. None of the neurons in the control ganglia were found to express Ki67 (data not shown).

To better characterize the proliferation of the new detected cell population and to define their differentiating pathway, many markers that characterized the earliest steps of the neuronal identity, from stem cell to neuroblast, were applied (Figure 8).

At day 3, small Ki67-positive cells surrounding neuronal profile coexpressed nanog, a marker for pluripotent stem cells (Figure 8(a)). Five days after crush injury, the same cell population surrounding the big neurons, some of which labeled with peripherin, was seen positive for sox-2, a marker for undifferentiated cells (Figure 8(b)). At the same time point, many BrdU-positive cells surrounding the DRG neurons also expressed nestin, a marker specifically expressed in neuronal progenitors (Figure 8(c)). Ten days after crush injury, many of the cells located peripherally to the ganglionic neurons were found to express NeuroD, a marker expressed in cells already committed to the neuronal lineage (Figure 8(d)).

A double labeling for nestin and either GFAP, a marker for immature glia, or S-100, a marker for mature glia, showed that, at day 5, some cells were found coexpressing nestin and GFAP (Figure 8(e)); however, none of the cells expressing nestin were found S-100-positive (Figure 8(f)).

**Figure 9:**
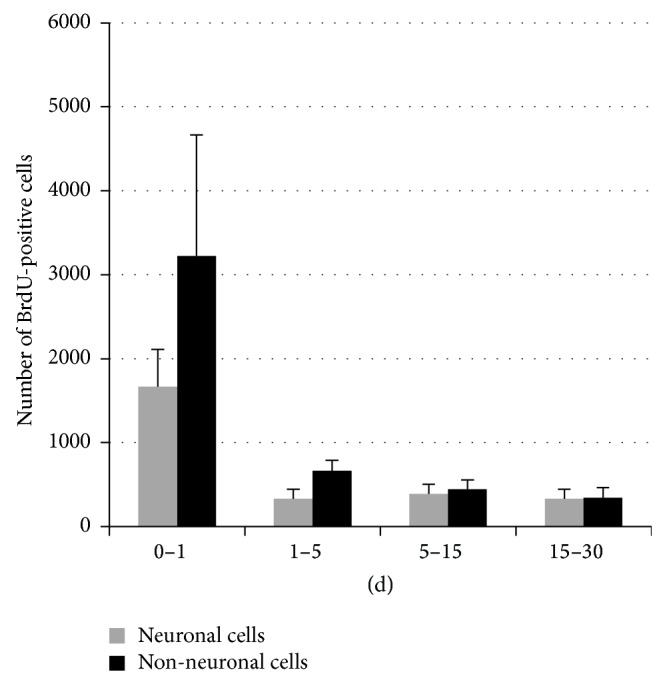
Histograms showing stereological analysis of BrdU-positive cells. The number of neuronal and nonneuronal BrdU-positive cells measured in each time window was divided by the days of BrdU treatment in order to predict the daily rate of cells which incorporate BrdU in the different time windows. (See Figure 2 for experimental design of BrdU injection.) All data are expressed as average ± standard error. The data were analyzed using both ANOVA and *t*-test analyses. ^**^ *P* ≤ 0.01; ^***^ *P* ≤ 0.001 versus 0-1 d group.

Finally, time course quantification of BrdU-positive nuclei threw light on the progression of DNA synthesis after crush injury. Daily rate of BrdU incorporation in the four time windows (Figure 9) showed that DNA synthesis, for both neuronal and nonneuronal cells, was maximal immediately after crush and decreases progressively along the postlesion time.

## 4. Discussion

The issue of adult plasticity has always been strongly debated if not denied by the neuroscience community according to Bizzozero's classification of neurons in the perennial cell category [36, 37]. Although the existence of neurogenic areas in the CNS has been commonly accepted [10], only few studies in the past 10 years have begun to come out with evidence of neurogenesis occurring in the peripheral nervous system. Apart from the only site in the PNS where neurogenesis has been undoubtedly documented and accepted, the olfactory neuroepithelium [38–42], the occurrence of adult neurogenesis in other sites of the PNS (dorsal root ganglia and autonomic ganglia) can be only postulated, since experimental data published so far are controversial.

The first evidence for the existence of neurogenesis and, hence, the presence of neural progenitors in DRG was based on counting methods [2, 3, 8, 43]. Although the findings were encouraging, the wide range of results obtained from different groups, due to different methods used to quantify neurons [13], never clarified whether plasticity actually occurs* in vivo* [8, 17]. Recently, new studies added data in support of adult neurogenesis in the PNS, both on nodose ganglion [44] and on DRG [27], after systemic capsaicin treatment. For the first time, here, we present evidence and quantification of a dramatic increase in the neuronal population belonging to the DRG in adult rats as a consequence of peripheral nerve damage and regeneration. Most of the previous studies applied stereology to thick vibratome or paraffin cut sections [23] which do not guarantee that all DRG neurons, especially the smaller ones, are identified. This might explain the disagreement of our results with those published by Degn et al. (1999) [45]. Since the physical disector method is based on the adequate recognition of morphological structures, as, for instance, the presence of the nucleus and the volume that the nucleus occupies, good histology and slice thickness are critical for a precise cell count. In this study, semithin sections (2.5 *µ*m) of resin-embedded T1 DRG were used therefore assuring high histological quality, no problems of artifacts, as, for instance, tissue shrinkage [46], and a precise cell count. Moreover, the analysis was done directly under light microscope at a high magnification to better recognize the presence of nuclei, thus discriminating countable from uncountable neurons.

The number of DRG neurons counted in the animals that underwent crush nerve lesion was persistently higher (42%) compared to controls and the data correlate with the volume of the DRG organ, which was also significantly increased. For a more scrupulous validation of our data, we also evaluated the total number of neurons in the contralateral ganglia harvested from the same animals that underwent crush lesion and control, respectively. We thus introduced an internal control to establish the range of variability among the neuronal population belonging to pairs of DRG. Partially in agreement with Ygge and coworkers [47], we found a small and statistically nonsignificant fluctuation in the number of DRG neuronal cells between left and right T1s; therefore, we conclude that a true increase in the number of neurons occurs in the DRG affected by peripheral nerve crush lesion in comparison to control groups. Moreover, the stereology perfectly correlates with morphological results, which showed* in vivo* the structure and the ultrastructure features of the newborn neurons.

Therefore, the data obtained using different experimental approaches allow us to state that the neuronal population of spinal ganglia after axonotmesis is far from being static; on the contrary, we document here an activation of the DRG cell populations that ends in a neuronal addition. This data are strongly supported by evidence of BrdU-labeled neuronal nuclei and the characterization of the morphological and immunocytochemical steps throughout the differentiating path of these new neuronal cells.

Stereological quantification of BrdU-positive cells showed that, as expected, DNA synthesis peaks at day 1 and progressively decreases after nerve damage for both nonneuronal and neuronal cells. Although it is known that posttraumatic DNA synthesis can increase as a consequence of the damage without necessarily being followed by cell division [48, 49], we believe that part of the BrdU labeling is due to proliferation of new neuronal cells, in accordance with previous literature [50]. Assuming that the BrdU incorporated by neurons at 1 day could reflect DNA repair, we still see an increase in BrdU-positive neurons until 30 days after injury in accordance with the stereological data.

Finally, to further support our hypothesis, we show that Ki67 was found expressed in a subpopulation of neurons. Due to the very restricted window of expression of Ki67, a quantification of Ki67-positive cells would probably lead to a result much less interesting than the qualitative result itself that shows an endogenous marker of proliferation, thus reflecting neurogenesis and not DNA repair, expressed in neuronal nuclei.

Although the possibility that proliferating cells are resident and/or infiltrating macrophages cannot be completely ruled out, the pattern of expression of precocious neuronal and glial markers, as shown by the immunocytochemical characterization, led us to hypothesize that the neuronal progenitors may originate from dedifferentiation of satellite cells. These neuronal progenitors, activated by an exceptional stimulus, for instance, the crush lesion of the peripheral nerves, may proliferate, differentiate into neuroblast cells, and then become new mature neurons. Therefore, we postulate a role for the DRG satellite cells in guaranteeing neuronal cell recruitment as a consequence of damage occurring in the peripheral nervous system.

## Figures and Tables

**Table 1:** Primary antibodies used for immunofluorescence.

Antibody characteristics	
Antibody	Manufacturer, catalog number, species, dilution.
a-Neurofilament	Sigma, St. Louis, MO, N014 Mouse monoclonal 1 : 200
a-Peripherin	Chemicon International, AB1530 Rabbit polyclonal 1 : 1000
a-S100	Sigma, St. Louis, MO, S2644 Rabbit polyclonal 1 : 600
a-GFAP	Sigma, St. Louis, MO, G9269 Rabbit polyclonal 1 : 500
a-Nestin	Sigma, St. Louis, N5413 Rabbit polyclonal 1 : 1000
a-SOX-2	Santa Cruz Biotechnology, Santa Cruz, CA sc-17320, goat polyclonal, 1 : 50
a-Ki67	Novocastra Laboratories, Newcastle, UK NCL-Ki67-MM1, mouse monoclonal 1 : 500
a-NeuroD	R&D System, Minneapolis, MN Goat polyclonal 1 : 500
a-Nanog	sc-33760, rabbit polyclonal, 1 : 1000
a-BrdU	Sigma, St. Louis, MO, B-2531 Mouse monoclonal 1 : 500
